# Feasibility and Acceptability of a Digital Patient-Reported Outcome Tool in Routine Outpatient Diabetes Care: Mixed Methods Formative Pilot Study

**DOI:** 10.2196/28329

**Published:** 2021-11-03

**Authors:** Soren E Skovlund, Lise Havbæk Troelsen, Lise Mellergaard Noergaard, Anna Pietraszek, Poul Erik Jakobsen, Niels Ejskjaer

**Affiliations:** 1 Department of Clinical Medicine, Aalborg University Aalborg Denmark; 2 Department of Endocrinology Aalborg University Hospital Aalborg Denmark; 3 Steno Diabetes Center North Denmark Aalborg University Hospital Aalborg Denmark

**Keywords:** patient-reported outcomes, diabetes, person-centered care, person-centered communication, dialog, mental health, self-management, collaborative care, value-based health care, mixed-methods, mobile phone, mHealth

## Abstract

**Background:**

Improvements in the digital capabilities of health systems provide new opportunities for the integration of patient-reported outcome (PRO) solutions in routine care, which can facilitate the delivery of person-centered diabetes care. We undertook this study as part of our development of a new digital PRO diabetes questionnaire and clinical dialog support tool for use by people with diabetes and their health care professionals (HCPs) to improve person-centered diabetes care quality and outcomes.

**Objective:**

This study evaluates the feasibility, acceptability, and perceived benefits and impacts of using a digital PRO diabetes tool, *DiaProfil,* in routine outpatient diabetes care.

**Methods:**

Overall, 12 people with diabetes scheduled for routine medical diabetes visits at the outpatient clinic were recruited. Purposive sampling was used to optimize heterogeneity regarding age, gender, duration, type of diabetes, treatment modality, and disease severity. Participants filled out a PRO diabetes questionnaire 2 to 5 days before their visit. During the visit, HCPs used a digital PRO tool to review PRO data with the person with diabetes for collaborative care planning. Participants completed evaluation forms before and after the visit and were interviewed for 30 to 45 minutes after the visit. HCPs completed the evaluation questionnaires after each visit. All visits were audio-recorded and transcribed for analysis. Data were analyzed using quantitative, qualitative, and mixed methods analyses.

**Results:**

People with diabetes found the PRO diabetes questionnaire to be relevant, acceptable, and feasible to complete from home. People with diabetes and HCPs found the digital PRO tool to be feasible and acceptable for use during the diabetes visit and would like to continue using it. HCPs were able to use the tool in a person-centered manner, as intended. For several people with diabetes, completion of the questionnaire facilitated positive reflection and better preparation for the visit. The use of the PRO tool primarily improved the quality of the dialog by improving the identification and focus on the issues most important to the person with diabetes. People with diabetes did not report any negative aspects of the PRO tool, whereas HCPs highlighted that it was demanding when the person with diabetes had many PRO issues that required attention within the predefined time allocated for a visit.

**Conclusions:**

The Danish PRO diabetes questionnaire and the digital tool, *DiaProfil,* are feasible and acceptable solutions for routine diabetes visits, and this tool may generate important benefits related to advancement of person-centered care. Further research is now required to corroborate and expand these formative insights on a larger scale and in diverse health care settings. The results of this study are therefore being used to define research hypotheses and finalize real-world PRO evaluation tools for a forthcoming large-scale multisector implementation study in Denmark.

## Introduction

### Background

Successful diabetes care requires a whole-person, collaborative care approach that focuses on an individual’s biological, psychological, and social health, well-being, functioning, values, preferences, and priorities [1].

Digital patient-reported outcome (PRO) solutions for clinical practice may help improve aspects of the care experience regarding person-centered chronic illness care [2] and potential outcomes of care [3]. PRO interventions may facilitate aspects of empowerment for people with diabetes through facilitation of reflective learning regarding (1) how diabetes affects one personally, (2) one’s preferred role in own care, (3) prioritization of own goals and taking an active role in developing action plans, and (4) structuring a process of ongoing experimentation and self-evaluation of action plan outcomes and efforts over time [4,5]. PRO tools can additionally function as support for health care professionals (HCPs) with regard to person-centered dialog, care planning, coordination of care, treatment decisions, treatment and outcome monitoring, psychosocial screening, and shared decision-making [6].

We developed a new digital tool, *DiaProfil,* to facilitate the use of PRO data by HCPs and people with diabetes in collaborative care visits using a formative, participatory design involving people with diabetes, family members of people with diabetes, and a multidisciplinary HCP team in all design phases [3]. Participatory research and user involvement help ensure that health care interventions are fit for the intended purpose [7-11], can be seamlessly integrated into care, and can deliver optimal public health impact [12-14]. We adopted these methods in the development of both the national PRO diabetes questionnaire and the PRO digital tool, and this pilot study was a part of the formative evaluation process.

*DiaProfil* provides a user-friendly mobile or internet interface for people with diabetes to complete a psychometrically designed, adaptive diabetes PRO questionnaire before their scheduled diabetes visits. This questionnaire can be completed via mobile phones, tablets, or PCs and provides a one-screen interactive overview (dashboard) of the PRO results for use by HCPs with the person with diabetes during the visit. The PRO dashboard is designed to make the PRO data available in a way that facilitates effective, collaborative, and action-orientated use of the data during the care visit. The aim is that HCPs and people with diabetes use the tool together to gain a common understanding of the overall perspective of the person with diabetes regarding their overall life with diabetes, priorities, and needs.

### Aims

This study aims to evaluate the feasibility and acceptability of the PRO diabetes questionnaire and the first viable version of *DiaProfil* in routine diabetes visits at an outpatient clinic and to undertake the initial exploration of the perceived benefits related to the use of PRO by people with diabetes and HCPs. The specific research questions were as follows:

1. How do people with diabetes experience the feasibility, acceptability, relevance, comprehension, and adequacy of topic coverage of the PRO questionnaire when used as intended in the context of a routine visit?
2. How do people with diabetes and HCPs experience the use of *DiaProfil* in connection with a routine diabetes visit in relation to feasibility, acceptability, perceived benefits, clinical utility, and challenges?
3. Specifically, do people with diabetes and HCPs experience the intended and hypothesized benefits of the PRO tool in improving patient participation and quality of the dialog?

As part of the formative process, this study additionally aims to facilitate the refinement of research hypotheses and finalize evaluation questionnaires for use in future real-world studies on the implementation and effectiveness of the *DiaProfil* tool in Denmark.

## Methods

This study was a formative, mixed-methods, single-arm, acceptability, feasibility pilot study that evaluated an office-based digital PRO tool intervention at an outpatient diabetes clinic.

### Recruitment

The eligibility criteria were age >18 years, diagnosis of type 1 or type 2 diabetes, diabetes duration of at least 1 year, and planned attendance for a routine visit at the diabetes outpatient clinic during the study period. Exclusion criteria were severe mental illness or major cognitive or language difficulties that would prevent the ability to fill out the diabetes questionnaire.

We used purposive sampling and consecutive recruitment to maximize the representation of type and duration of diabetes, age, gender, treatment regimen, and disease severity. Eligible participants were identified by the study nurses from the electronic booking system in the diabetes clinic and invited to take part in the pilot study by telephone. The study was described as a pilot test of a diabetes questionnaire designed to help improve the quality of diabetes visits. All participants signed informed consent before study enrollment.

### Study Design

The study was approved by the local institutional review board and deemed out of scope for the ethical review board because of the absence of a clinical treatment or intervention and limited risk.

The study design and intervention included the following:

1. Each participant received a secure email with a weblink for mobile or web-based access to complete informed consent, the PRO diabetes questionnaire, and an evaluation questionnaire about the PRO questionnaire. The participant was asked to complete this questionnaire 2-10 days before their scheduled visit. If it was not possible to do it at home, the person with diabetes was encouraged to contact the clinic to arrange on-site completion.
2. HCPs were able to immediately access PRO results once completed by the person with diabetes but were asked to only access results immediately before the visit to mimic routine diabetes care.
3. At the visit, HCPs used the *DiaProfil* PRO dashboard with the person with diabetes to jointly review PRO results and plan care collaboratively around the priorities of the person with diabetes. Each visit was audio-recorded and transcribed verbatim.
4. The HCPs and people with diabetes completed the evaluation questionnaires immediately after the visit about their experiences using PRO. HCPs filled out a web-based form describing any issues, errors, or concerns relating to the PRO results and how they were displayed using algorithms.
5. People with diabetes were interviewed for 30-45 minutes using a semistructured interview guide right after the diabetes visit by a researcher not involved in care for that person with diabetes. The PRO dashboard and questionnaire results were available in the interview to facilitate a detailed discussion with the person with diabetes about any feedback to the individual’s PRO results, as shown on the PRO dashboard. It was emphasized upfront to people with diabetes that their feedback via questionnaires and interviews was kept strictly confidential and would not be shared with the clinical care team.

### The PRO Diabetes Intervention

**Figure 1:**
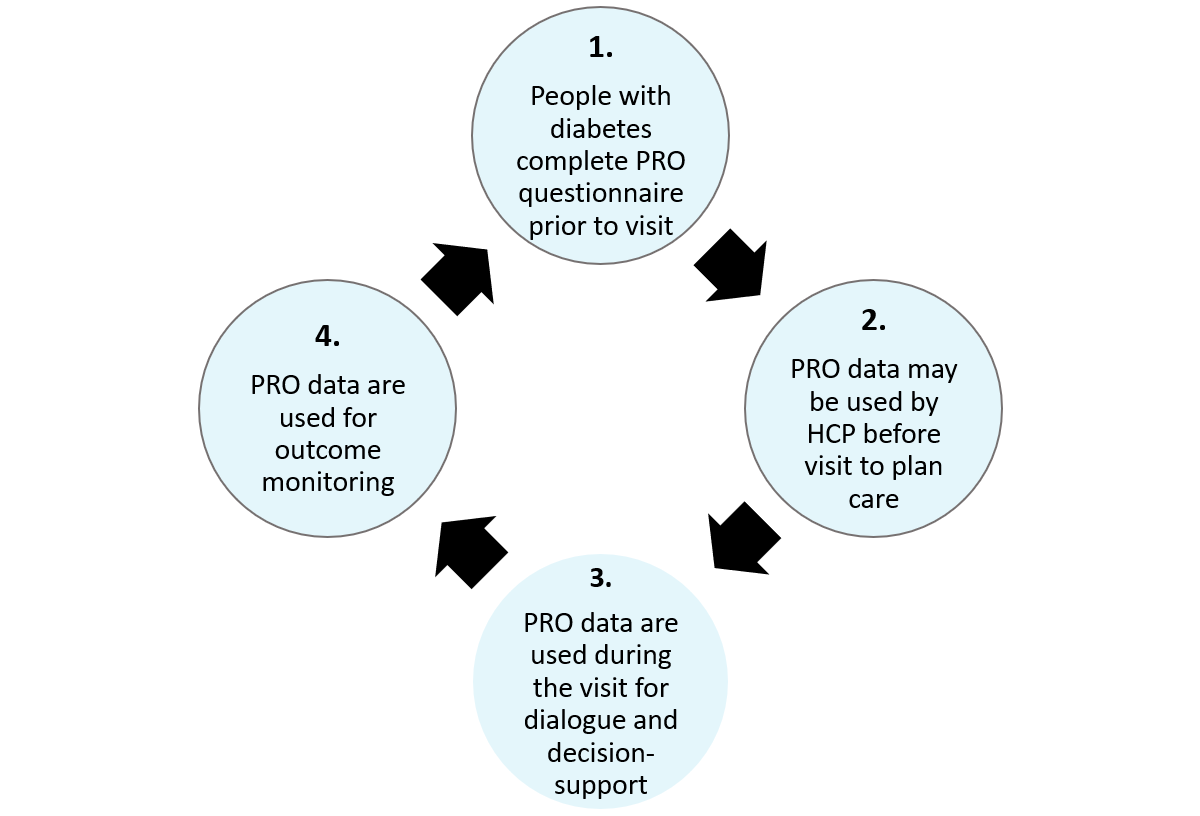
Basic steps of the patient-reported outcome diabetes intervention in clinical practice. PRO: patient-reported outcome.

The aim of the PRO diabetes intervention was to increase the active participation of people with diabetes in their own care and improve the quality of the dialog between people with diabetes and HCPs, and overall care quality by focusing on optimizing value for people with diabetes [3]. The basic steps of the PRO diabetes intervention are shown in Figure 1. The digital tool *DiaProfil* was used by people with diabetes to complete the PRO questionnaire and by HCPs to manage PRO data and use the PRO data during visits.

People with diabetes completed the PRO diabetes questionnaire by phone, tablet, or PC using *DiaProfil* at home 2-10 days before the visit. The diabetes questionnaire consisted of 33-71 items (depending on the activation of branch logic) that measured health, life situation influencing diabetes, general and diabetes-specific social support, psychological well-being, depression, symptom distress (neuropathy pain, cardiovascular symptoms, gastrointestinal symptoms, sexual dysfunction, sleep difficulties, and foot problems), daily life with diabetes, worries about diabetes, confidence in diabetes self-management, blood sugar regulation (including hypoglycemia and blood sugar stability), medical experience and satisfaction, access to HCPs, priority issues for support, and preferred topics to discuss. The HCPs used the PRO dashboard in *DiaProfil* with the person with diabetes during the visit to review the person’s priorities and issues and collaboratively plan care. The HCPs were recommended to review the PRO dashboard in advance, share the screen for mutual viewing, explain the PRO dashboard and the color-coding, maintain nonverbal communication and eye contact, use open-ended questions and active listening to prompt more information and confirm findings, and cover all flagged PRO issues. Our recommendations for person-centered use of the PRO tool were quite similar to recently recommended strategies for person-centered communication when using PRO data in clinical practice in other studies [15].

### The Development and Design of the Digital PRO Diabetes Tool: DiaProfil

This formative study is a part of the participatory development process of the digital PRO tool *DiaProfil* by the VBHC-PRO-DIA (Value-Based Health Care and PRO in Diabetes) research team and was conducted from 2018 to 2020. We developed *DiaProfil* as a new tool to facilitate the coordinated use of the national PRO diabetes questionnaire, also developed as part of this project, in different health care settings to improve person-centered, value-based diabetes care. The PRO tool measures diabetes outcome constructs previously established as important to people with diabetes and HCP in Denmark [16].

Each PRO item or scale score is shown on the *DiaProfil* HCP dashboard using a color defined by a predefined scoring algorithm. Green indicates that there is likely no problem for the person with diabetes, yellow indicates a possible issue of concern for the person with diabetes that should be considered, and red indicates that there appears to be a problem that the HCP and the person with diabetes should make sure to review and address.

The interface for people with diabetes includes a user-friendly digital interface for questionnaire completion, which was developed and tested with people with diabetes using an iterative participatory process with user-testing to optimize user-friendliness. Only one question is depicted on the screen at a time to facilitate ease of use and lower cognitive burden.

The dashboard provides a one-screen instant overview of PRO results by presenting the results in 9 main themes. On the right side of the screen, the person with diabetes’ own priorities for self-management support and topics to discuss are flagged for use as a starting point for the dialog. By clicking or touching the screen, the HCP can access dialog tips, information resources, local treatment, and referral information relevant for each PRO output.

**Figure 2:**
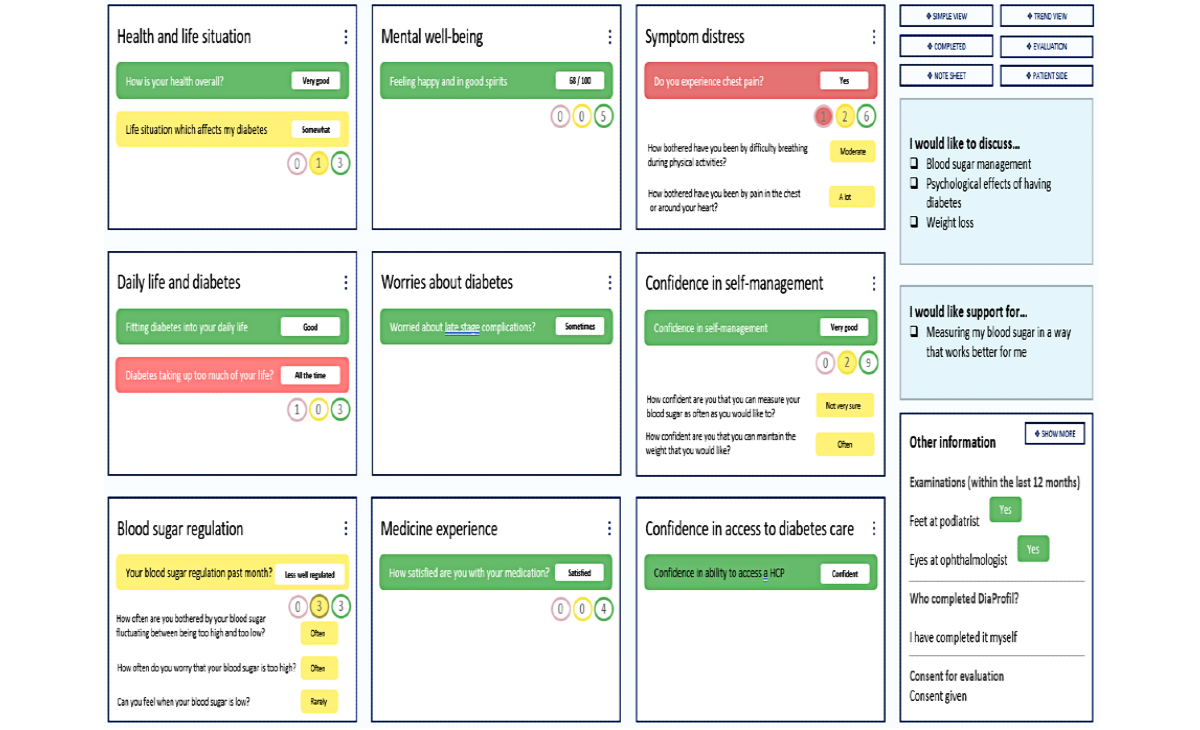
Screenshot from the digital patient-reported outcome diabetes tool, *DiaProfil*. Only examples are shown. The text is abbreviated. The figure is only intended to illustrate the design of the dashboard. PRO: patient-reported outcome.

Figure 2 shows a screenshot of the PRO dashboard with examples of how the results may be shown. Depending on individual PRO results, a variable level of information is shown on the screen.

### Involvement of People With Diabetes in the Research Process

A panel of people with diabetes who represented the target group for the PRO intervention were involved as partners in this study to ensure that the perspective of people with diabetes was considered at all research phases [17]. This was important to ensure relevance of the research and research questions to people with diabetes [18]. A total of 2 adults with type 2 diabetes and 3 with type 1 diabetes were part of this panel. Of these 5 participants, 3 (60%) were women, and 2 (40%) were men. The participants’ age ranged from 30s to 70s, and they had different levels of disease burden. Approximately 60% (3/5) had extensive experience representing unmet needs and perspectives of people with diabetes beyond personal experiences and 40% (2/5) had some previous experience. One was also a health professional and was able to also consider the perspective of HCPs. The main method of involvement was group work meetings that involved both the user panel and members of the multidisciplinary clinical research team. This group focused on reviewing and cocreating the study aims, study design, sampling strategy, and evaluation questionnaires. In addition, this group worked with hands-on user-testing and co-designing the *DiaProfil* app tool and interface for people with diabetes. The user panel also contributed to the scientific development of parts of the PRO diabetes questionnaire.

### Data Collection

Clinical charts (HbA_1c_, cholesterol, blood pressure, and complication data), sociodemographic data, and treatment data (age, gender, duration of diabetes, type of diabetes, medical therapy, and technology use) were collected from all study participants using chart reviews and questionnaires. PRO and evaluation questionnaires were administered to people with diabetes and HCPs using the *DiaProfil* platform. People with diabetes completed evaluation questionnaires before and after their consultation, and HCPs completed the questionnaires after the visit. Questionnaires were purposely based on qualitative data collected from workshops held with people with diabetes and HCPs as part of the development of the PRO questionnaire.

An overview of the evaluation questionnaires is shown in Textbox 1. Generic items were designed to evaluate the quality of autonomy-supportive and person-centered communication [19,20]. The semistructured interview guide was designed based on research questions for the study and qualitative data from involvement of people with diabetes during the intervention development process. The interview guide addressed the following main elements: (1) motivation to participate; (2) experience related to PRO completion before the visit; (3) experience related to the use of PRO results and the dashboard during the visit; (4) experience of any problems or disadvantages because of PRO; (5) comprehension, acceptability, and face validity of items and scoring algorithms; and (6) overall judgment, perceptions, attitudes, and recommendations regarding future use of the intervention.

All consultations and semistructured interviews were audio-recorded and transcribed verbatim. Four consultations were observed in person by a clinical diabetes psychologist for HCP supervision purposes to complement informant perspectives and assess any potential risks related to the way psychological issues are identified, addressed, followed up on, and reacted to.

**Evaluation questionnaires.**

**The Danish Patient-Reported Outcome (PRO) diabetes questionnaire (33-71 items)**

- Completed by people with diabetes at home in advance of the visit. Evaluates general health and life situation, well-being, depression, symptom distress, annual check of feet and eyes, daily life with diabetes, worries about diabetes, confidence in managing diabetes, blood sugar regulation and hypoglycemia, medicine experience, access to health care professionals (HCPs), priority areas for self-management support, and preferred topics to focus on for the visit.

**PRO diabetes questionnaire Evaluation Pilot Questionnaire for People With Diabetes (PRO-EVAL-P-1A; 4 items)**

- Completed by people with diabetes immediately after the PRO questionnaire; evaluates perceptions of (1) relevance, (2) difficulty, (3) comprehension, (4) topic coverage/comprehensiveness, (5) acceptability, and (6) item-specific issues.

**PRO Consultation Evaluation Pilot Questionnaire for People With Diabetes (PRO-CON-EVAL-P-1A; 14 items)**

- Completed by people with diabetes immediately after the PRO questionnaire diabetes visit; evaluates perceived (1) support for autonomy and person-centered communication; (2) PROs impact on dialog, role, and care; (3) potential negative impacts; (4) face validity of scoring algorithms; (5) interest in continued use and advocacy; and (6) suggestions for improvement.

**PRO Consultation Evaluation Pilot Questionnaire for HCP (PRO-CON-HCP-1A; 10 items)**

- Completed by HCPs immediately after the PRO questionnaire diabetes visit; measures perceived (1) quality of visit; (2) PROs impact on dialog, roles, visit outcome; (3) challenges with the use of PRO; (4) satisfaction and interest in future use; and (5) clinical validity of items and algorithms.

### Data Analysis

Quantitative data (blood tests, sociodemographic, PRO questionnaire, and Likert scale evaluation questionnaire) and qualitative data (consultation and interview transcripts, free-text evaluation responses, and notes from debriefing meetings) were analyzed in SPSS Statistics 25.0 for Windows (IBM Cooperation) and NVIVO 12.0 (QSR International), respectively. Primary quantitative evaluation data were presented descriptively. The main qualitative analysis used a simple stepwise coding process adapted from thematic analysis and a phenomenological and combined inductive and deductive approach [21-23]. The aim was to evaluate perceived feasibility, acceptability, benefits, and drawbacks among people with diabetes and HCPs while examining the extent to which PRO was perceived as having an impact on the participation of people with diabetes, quality of dialog, overall care visit, and follow-up. The steps used by the qualitative researcher included (1) all data being reviewed and iteratively coded using the research questions as topic structure; (2) all codes being analyzed for duplicity, relationships, and hierarchical structures; (3) key categories and themes being identified; (4) robustness of each category or theme being checked by reviewing each against all raw data; and (5) results being continuously checked and discussed with the multidisciplinary clinical team, multiple coders, and a panel of people with diabetes. The analysis included an exploratory element to assess whether benefits or disadvantages were highlighted, which did not fit within the predefined research questions. Consultation transcripts were analyzed using a predominantly semantic content analysis approach [24] to assess selected aspects of fidelity, feasibility, and acceptability regarding the use of PRO during the visit. A codebook was developed based on a review of all transcripts with 4 main coding categories: (1) HCP use of the PRO dashboard (sharing and use of open-ended prompts), (2) response of the person with diabetes when the HCP prompted a specific PRO result (validating its relevance), (3) follow-up action in response to prompted PRO topics (eg, problem-solving dialog, referral, therapy change, education, and new appointment), and (4) PRO topic code (PRO item, scale, or construct). One researcher coded all transcripts to establish the codebook, and 3 students coded 3 interviews each to compare scoring and identify codebook ambiguities. Work meetings were conducted to refine the codebook until concordance in coding was established. Qualitative data were used for the explanatory analysis of the quantitative evaluation questionnaire data as a mixed-methods design.

## Results

### Overview

The characteristics of the 12 people with diabetes enrolled are shown in Table 1. A good variance was achieved in relation to gender, type of diabetes, age, duration of diabetes, therapy and treatment modality, complications, and comorbidity burden. The HCPs were 2 senior diabetes nurses and 2 senior diabetes physicians employed at the Ambulatory Diabetes Clinic at Aalborg University Hospital. All HCPs (3/4, 75% women and 1/4, 25% men) had >5 years of diabetes care experience, and all had had some previous involvement with the design of the PRO diabetes tool.

**Table 1:** Characteristics of study participants (N=12).

Characteristics			Values
Gender (female), n (%)			7 (58)
**Type of diabetes, n (%)**			
	Type 1	8 (67)	
	Type 2	4 (33)	
Age (years), median (range)			56.6 (24-79)
Duration (years), median (range)			19.5 (2-50)
**Device used, n (%)**			
	Insulin pen	7 (58)	
	Insulin pump	2 (17)	
GLP-1^a^, n (%)			3 (17)
Tablet, n (%)			1 (8)
**Number of comorbidities**			
	Mean	1.58	
	Median (range)	1 (0-8)	
**Number of complications**			
	Mean	1.3	
	Median (range)	0.5 (0-4)	
**Outcome variables, mean (SD; range)**			
	HbA_1c_^b^ (mmol/mol)	85.8 (20.7; 61-113)	
	Health (SF-1^c^; score range 1-5)	3.0 (0.7; 2-4)	
	Well-being (WHO-5^d^; score range 0-100)	60.4 (20.7; 20-96)	
	Diabetes-specific distress (PRO^e^ Diabetes Questionnaire–Negative Impact of Diabetes Scale, three-item distress scale; score range 0-100)	41.0 (14.0; 8-58)	
	Number of PRO topics flagged for action (scored with a *red* flag)	3.8 (2.7; 0-10)	
	Number of PRO topics flagged for action (scored with a *yellow* flag)	4.8 (3.0; 1-11)	
	Total number of PRO topics flagged for action (yellow and red topics and additional topics selected by people with diabetes)	14.3 (6; 4-26)	

^a^GLP-1: glucagon-like peptide-1.

^b^HbA_1c_: glycated hemoglobin A_1c_.

^c^SF-1: global health item of the Short-Form Health Survey.

^d^WHO-5: World Health Organization–Five Well-Being Index.

^e^PRO: patient-reported outcome.

### Quantitative Data Results

#### Evaluation of the PRO Diabetes Questionnaire by People With Diabetes

The results from the quantitative evaluation of the PRO questionnaire by people with diabetes are shown in Table 2.

All the people with diabetes were positive about the relevance of the questions for their diabetes, except 1 person with diabetes who indicated a moderate negative appraisal. All people with diabetes found it easy to complete the entire questionnaire. Of the 12 participants, 6 (50%) indicated no items, and 6 (50%) indicated *one or few items* of the PRO questionnaire were difficult to understand. The specific items were reviewed with people with diabetes in interviews as part of the formative questionnaire validation and design. Of the 12 participants, 10 (83%) indicated that no important topics were missing from the questionnaire, and 2 (17%) mentioned topics of *atypical diabetes type* and *more on how to access pump technology* as desired topics. Both interview and questionnaire data confirmed that people with diabetes felt the color-coded display of their PRO data, including specific cut-off thresholds in *DiaProfil*, provided a valid and helpful picture of their situation. Furthermore, HCPs provided separate confirmations of the validity of the scored PRO outputs for each person with diabetes after each visit.

**Table 2:** Quantitative evaluation of the patient-reported outcome diabetes questionnaire by people with diabetes (N=12)^a^.

Response options				Value
**How relevant did you find the questions to be for your diabetes care?**				
	Mean score (SD)			3.5 (0.7)
	**Negative appraisal^b^, n (%)**			
		1	0 (0)	
		2	1 (8)	
	**Positive appraisal^b^, n (%)**			
		3	4 (33)	
		4	7 (67)	
**How difficult or easy was it for you to complete the questionnaire?**				
	Mean score (SD)			3.6 (0.7)
	**Negative appraisal^b^, n (%)**			
		1	0 (0)	
		2	0 (0)	
	**Positive appraisal^b^, n (%)**			
		3	6 (50)	
		4	6 (50)	
**Were any items difficult to understand? n (%)**				
	No			9 (75)
	Yes, one or a few			3 (25)
	Yes			0 (0)
**Did you miss any topics missing? n (%)**				
	No			10 (83)
	Yes			2 (17)

^a^Shows the responses of people with diabetes to the PRO-EVAL-P-1A pilot evaluation questions immediately after completing the PRO questionnaire.

^b^A score of 1 and 2 reflects a negative appraisal, and 3 and 4 represents a positive appraisal.

#### Questionnaire Evaluation of the Use of PRO During the Visit by People With Diabetes

The mean single-item scores by item for the primary evaluation questions are shown in Table 3.

All 12 people with diabetes rated the person-centered autonomy-supportive communication style of the HCPs positively, with mean scores ranging between 4.5 and 4.8, with a score range of 1-5. People with diabetes felt that their HCPs were focused on their priorities, encouraged them to speak, and made them feel comfortable talking about their needs, and all felt they got the care and advice that they had hoped for. In order of decreasing positive rating, people with diabetes expressed high interest in continued use, that PRO should be offered as standard care to all, that PRO helped focus on what was most important to them, that PRO helped focus the conversation on what mattered most to them, that they would like to use PRO in their future care, that they felt better prepared for the visit, and that the PRO dashboard provided a good picture of their current diabetes situation, needs, and priorities. There was only 1 person with diabetes who indicated a moderate degree of being uncomfortable or having a problem related to the use of PRO. During the interview, where answers were debriefed, she explained that she had had a bad day, was feeling very distressed because of diabetes, and had not felt the HCP understood her issues as they were raised. Despite this, she was very positive about the PRO tool and did not attribute the problem to the tool.

**Table 3:** Quantitative evaluation of the use of patient-reported outcome (PRO) during the visit by people with diabetes (N=12)^a^.

Items		Single-item score^b^, mean (SD)
**General dialog evaluation**		
	Did the HCP^c^ make you feel comfortable talking about the topics that you needed to?	4.8 (0.6)
	Did the HCP give you the treatment, advice, referral, or other assistance that you needed?	4.6 (0.5)
	Did the HCP focus on what is most important to you?	4.5 (0.9)
	Did the HCP encourage you to give input or ask questions?	4.5 (0.7)
**Evaluation of influence of use of PRO**		
	Interested in using PRO in future care	4.9 (0.3)
	PRO should be part of standard care	4.8 (0.4)
	The *DiaProfil* dashboard helped me talk about the important things	4.7 (0.3)
	HCP was more or less prepared because of use of PRO	4.4 (0.9)
	I felt more or less prepared because of use of PRO	4.4 (0.9)
	PRO dashboard gave a good picture of my situation, needs, and priorities related to my diabetes	4.3 (1.0)
	Any problems or uncomfortable experiences related to the use of *DiaProfil* in the dialog? If so, which?	1.3 (0.6)

^a^Mean single-item scores of evaluation by people with diabetes of use of PRO after the visit using the PRO-CON-EVAL-P-1A-pilot questionnaire.

^b^Score range is 1-5, except for the last item, which is 1-3.

^c^HCP: health care professional.

#### Results of the Qualitative Analysis of Interviews With People With Diabetes

The main themes identified from the qualitative analysis of the interviews are shown in Table 4, with illustrative quotes.

People with diabetes expressed that they felt the PRO questionnaire covered all relevant issues, was straightforward to fill out, and facilitated positive self-reflection. In line with the questionnaire evaluations, approximately half of the people with diabetes reported minor issues with understanding one or a few items.

Most people with diabetes expressed that they found it positive to complete the questionnaire at home in advance, as it helped them know what the conversation would be about at the visit. Filling out the questionnaire made the people with diabetes feel reassured that they would remember and get to talk about their priority issues with their HCP. This was important as several people with diabetes expressed frustration that they would often forget to talk about the issues that mattered to them during visits. A family member who participated with a study participant explained the following:

It is a nice thing to fill it out at home, and the HCP is also better prepared for what you want to ask about. I would like to have this every time.

Several people with diabetes had a positive personal experience filling out the questionnaire. One person with diabetes said the following:

When I sat down with the questionnaire, I had some time for it, and I felt really positive and surprised. Because it went straight in and touched on some issues where I had to feel and actually remove the shutters and relate to it. ”How am I doing? If I am totally honest, how is it going with this?“ It is easy to say, ”well it’s going to be fine.“ Let’s just keep going as usual. So, in this way it was really an eye-opener for me.

Only one person with diabetes expressed an uncomfortable situation related to the use of PRO during the visit, as noted earlier, and it was clarified in the interview that the person with diabetes did not attribute the issue to the PRO tool but attributed it to not feeling fully understood by the HCP.

**Table 4:** Analysis of semistructured interviews with people with diabetes.

Theme and subcategories				Quotes for illustration
**Perception of the questionnaire**				
	**Easy and manageable to fill out**			
		Easy to do A positive experience No or few items difficult to understand	“I felt it was easy and I think it was well laid out with 5 response options every time–and I just felt it was simple”	
	**Relevant comprehensive questionnaire**			
		All questions relevant No key topics missing 360° coverage is good	“It gets into all the issues, which I think is good because it makes you think about stuff you might not otherwise have”	
**Benefits related to filling out the questionnaire**				
	**Feel better prepared**			
		Self-reflection and self-insight	“It motivated me hugely to get some things on the table that I have been needing to talk about but closed my eyes to because I tend to just say things are 'kind of fine'?”	
		Comfort knowing own priorities will be addressed in the visit	“Many times I feel I forget when I get to the visit, shoot, this or that I forgot to ask about when you sit there–and when you leave you haven’t asked about what you needed. So, this is really super this tool”	
**Experience of the process of use of PRO^a^ in-visit**				
	**HCP^b^ uses PRO in pleasant or good way**			
		PRO screen was easy and intuitive in visit	“I think it was great to see [DiaProfil screen]–it made sense; red is bad, yellow is less bad and green that is perfect-ish, right?–I think it gave a good picture [of my situation] and it was easy for me to grasp it”	
		HCP talked about PRO results in a pleasant way	“I liked the way that she [HCP] went at it right away. She made me actually feel reassured by showing me the screen and just mentioning e.g. there was this box with a mental issue”	
**Benefits of PRO during the visit**				
	**Better, more meaningful care visit**			
		HCP is better prepared	”It felt very nice [that the nurse had seen my PRO answers], because I am thinking if she has read it through she might see some connections between my issues – makes sense.“	
		Cover new important topics that matter to me	“We covered some other topics than normal, because I usually just get the numbers [blood test results] and sometimes gets measured and weighted and then go home again. It got more personal. And I think that was awesome.”	
		Easier to talk about difficult or sensitive issues	”It was a bit easier to sit at home and write that I actually would like to get some help to stop smoking than to sit down here–now it’s out in the open without having to say it face to face. It also makes it easier for the HCP I think–you can maybe get to talk about some of the difficult topics that you wouldn’t just sit there and say.“	
		Getting better care	“I get better help by answering these questions”	

^a^PRO: patient-reported outcome.

^b^HCP: health care professional.

#### HCP’s Evaluation of the Use of PRO During the Visit

The main results from the HCP questionnaire evaluation of the visits are shown in Table 5. HCPs rated the general quality of the dialog positively, felt they were able to cover all the issues that were important to them, and were satisfied with the clinical outcome. In one case, the HCPs noted general dialog difficulties; however, this was reported by the HCP to be unrelated to PRO and caused by the fact that the person with diabetes had mild cognitive impairment and language difficulties. In order of decreasing positive ratings, HCPs were satisfied with the use of PRO, highly interested in continued use of the tool, and felt that all relevant topics were covered by the PRO questionnaire. HCPs reported moderate improvement in preparedness and active engagement among people with diabetes during the visit. HCPs were neutral or marginally positive about the ability of PRO to uncover previously unknown clinical challenges of people with diabetes, and in 50% (6/12) of visits, HCPs highlighted some challenges related to the use of PRO during the visit.

**Table 5:** Results of questionnaire evaluation of the use of the patient-reported outcome (PRO) diabetes tool by health care professionals (N=12)^a^.

Item content^b^		Mean single-item score (SD)
**General dialog evaluation**		
	How would you rate the overall quality of the dialogue with this patient?	4.25 (1.1)
	Did you cover all the topics in the visit that were important to you from a clinical perspective?	4.4 (0.7)
**Evaluation of influence of use of PRO**		
	How interested are you in using *DiaProfil* in its present form in your future diabetes consultations?	9.25 (1.0)
	How satisfied or dissatisfied were you overall with use of *DiaProfil* in the consultation?	4.6 (0.7)
	Was the person with diabetes was more or less prepared for your dialog due to answering the PRO questionnaire?	3.75 (0.75)
	Do you feel that your use of *DiaProfil* changed your role in the conversation?	3.5 (1.0)
	Did you experience that the person with diabetes got to speak more or less during this visit due to use of *DiaProfil?*	3.6 (0.5)
	Did DiaProfil make you aware of clinically relevant issues for this patient you were not aware of before?	2.67 (1.3)
	Did you experience challenges due to the use of *DiaProfil* during the visit?	2.33 (1.4)

^a^Mean single-item scores of health care professional evaluations of use of patient-reported outcome in the visit (PRO-CON-EVAL-P-1A-pilot).

^b^Score range is 1-5. Score of 1-2: negative; 3: neutral; and 4-5: positive (except for the question on interest in continued use, which has a score range of 1-10).

### Results of the Analysis of HCPs’ Free-Text Evaluations of Use of PRO in the Visit

Themes and illustrative quotes from the HCP’s evaluation of the visits are shown in Table 6. HCPs felt that PRO helped focus the dialog during most visits, and it was mentioned that the fact that people with diabetes had reflected on issues in advance contributed to this. Generally, HCPs did not indicate that PRO revealed new clinical treatment insights; however, in half of the visits, PRO placed attention on the fact that clinically relevant issues were of specific importance to their patients, with sleeping difficulty, erectile dysfunction, cardiovascular symptoms, worry about late-stage complications, foot problems, and psychological issues being problems specifically mentioned. The PRO tool was noted to provide structured insight, especially into the psychological factors at play. HCPs did not report any technical or functional issues related to using the digital tool but reported a number of challenges in half of the visits, reflecting in large part the fact that the HCPs were new to using the tool in a routine visit and there was no detailed manual for combining PRO and clinical data during the visit. Key issues raised by HCPs were how to handle the dialog if the person with diabetes had a lot of PRO topics requiring attention and that it could be difficult to find the right balance between traditional clinical tasks and the new PRO topics. Another issue that was raised was that some symptoms flagged in the PRO dashboard were not always related to diabetes or had already been acted upon by another HCP, so the value of raising these issues appeared unclear to the HCP.

**Table 6:** Analysis of open-ended text evaluations by the health care professional after each visit.

Main themes		Quote or case example
**Benefits of use of PRO^a^ in the visit**		
	The person with diabetes was better prepared due to self-reflection in advance	“I tend to ask a lot of questions if the patient does not say so much. In this case the patient had already reflected and prioritized which allowed us to focus on this instead of ‘shooting in the blind’.”
	Better able to set agenda in line with patient priorities	“I distributed the available time better, focused on the problems of the patient, listened more”
	New insights about which topics are important to the person with diabetes.	New topics identified as important to the person with diabetes included worry about complications, foot wound, erectile dysfunction, sleep, pain, and barriers in life situation to managing diabetes.
**Challenges regarding use of PRO in the visit**		
	Managing the conversation when there are many flagged PRO topics	“Dialogue would have been better if we had had more time to wrap up the various problem areas identified” “There were so many answers to consider that it extended the visit.”
**Not all PRO outputs were relevant to act on**		
	Red score on pain	A person with diabetes scored red on pain, but it was because of arthritis pain that was already treated and addressed in other care setting.
	Red score on low well-being	A person with diabetes scored red on low well-being, but it was because of life issues unrelated to diabetes.
	Uncertainties regarding use	Unsure how to handle a discrepancy between a PRO score and what the person with diabetes says in the visit.

^a^PRO: patient-reported outcome.

### Content Analysis of Transcripts of Diabetes Visits

The results of the content analysis of the audio recordings of the visits are summarized in Table 7. HCPs generally used the PRO dashboard as intended for dialog support (albeit in different ways and to different degrees). As there was no video recording of the visits, we could not make precise assessments of when and how the PRO dashboard was used to prompt each individual topic. This was especially difficult as both the person with diabetes and HCP would view the screen while talking. Overall, with few exceptions yellow or red colored topics and topics requested by the person with diabetes on the PRO dashboard questionnaire were prompted or acted upon during the visit by the HCP. Several topics were only briefly mentioned; however, they were reviewed and considered for relevance. The key topics that were shown to be both flagged as a potential problem area on the PRO dashboard, prompted in the visit by the HCP, confirmed as relevant by the person with diabetes and followed-up on by the HCP are depicted in Textbox 2.

Many PRO topics prompted by the HCPs were validated as relevant by the person with diabetes and, for the most part, resulted in relevant follow-up dialog and, in some cases, action. However, in line with HCP evaluations, there were several instances where topics raised pertaining to, for example, symptoms unrelated to diabetes were found not to lead to concrete action or follow-up plans. By cross-matching the topics discussed during the visit with the PRO data, we identified some individual errors because of mistakes by people with diabetes during questionnaire completion; however, we could exclude any structural PRO assessment problems and could confirm the clinical validity and utility of the PRO outputs. Observations of a subset of consultations by a clinical diabetes psychologist provided additional reassurance that it was possible for HCPs to incorporate PRO data in a person-centered manner into the dialog. Observations provided input to our future person-centered training for the use of PRO, especially regarding identifying and clarifying previously undetected psychosocial problems.

**Table 7:** Results of analysis of audio-recording transcripts of patient-reported outcome (PRO) diabetes consultations (N=12).

Category		Case	Result, n (%)
**Fidelity of in-visit use of PRO**			
	HCP^a^ used open-ended questions to prompt people with diabetes about a flagged PRO topic or result	“Is this something that you recognize?” [HCP] ”Here it says something about pain in your feet?” [HCP]	12 (100)
	HCP showed and explained the PRO dashboard	“What you answered in the questionnaire is shown on this screen.” [HCP] “If it is green it is all good, like a traffic signal, right?” [HCP] “It is like a traffic light. Yellow is something to be attentive to if there might be something to talk about.” [HCP]	10 (83)
	HCP explicitly used a PRO to set visit agenda	“And then there is red which we definitely should talk about...” [HCP] Case example: “Is it things that bother you?” [HCP]; “Yes” [person with diabetes]; “Ok then it makes sense we start out with this” [HCP]	8 (67)
	All flagged PRO topics were mentioned during the visit.	“You have indicated you feel that diabetes takes up too much of your daily life?” [HCP]	12 (100)
**Use of PRO in the visit**			
	At least one PRO topic was prompted by the HCP, validated as relevant by the person with diabetes, and followed up with action^b^	Case example: PRO topic flagged: ”Not confident in ability to get contact with HCP if needed”. HCP is prompted with open question. Person with diabetes validates result. HCP is informed about contact options and handed out a diabetes telephone hotline leaflet.	12 (100)

^a^HCP: health care professional.

^b^Actions may include follow-up dialog, advise, treatment, education, self-help resources, care planning, and referrals.

**Topics where cases of intended clinical use of the patient-reported outcome tool were identified in this study.**

**Topics**

- Life situation impacting diabetes
- Psychological well-being
- Daily life with diabetes
- Worry about complications
- Blood sugar regulation and hypo/hyperglycemia worry
- Diet, carbohydrate counting, and smoking
- Medicine experience
- Symptom distress: Sleep, sexual function, foot, neuropathic pain, gastrointestinal, and cardiovascular
- Confidence in access to health care professionals for diabetes

### Mixed-Methods Analysis

#### Overall Results

Qualitative and PRO data were compared with questionnaire evaluation data to verify the findings and establish robust overall findings regarding feasibility, acceptability, and perceived benefits. Both questionnaire and qualitative data confirmed that the PRO questionnaire and the *DiaProfil* tool were acceptable, valid, appropriate, and effective in improving people with diabetes’ preparation for the visit and the quality of the dialog.

Both questionnaire data and interviews for people with diabetes and visit transcripts supported that HCPs used the PRO dashboard in the intended person-centered way and achieved a more focused and relevant dialog. The HCP questionnaire and open-ended text confirmed general satisfaction with the use of PRO tools.

Some people with diabetes had a high number of flagged PRO topics in this version of the tool, and some PRO-flagged topics were not relevant for the HCP to act on in the specific visit; however, people with diabetes were satisfied with the use and did not report concerns or negative experiences related to this. We used a multi-informant mixed-methods approach to analyze negative outliers in the questionnaire data. As an example, we looked at the only person with diabetes who had rated the relevance of the PRO diabetes questionnaire as somewhat not relevant, whereas the 11 others had rated it relevant. The person with diabetes had had diabetes for >13 years, was resourceful and empowered in relation to diabetes, and had seen the same physician for several years. The person with diabetes’s *DiaProfil* dashboard was largely *green* but with flagging of pain, sleep, and blood sugar measurement. She indicated that as she was doing fine and knew what she wanted to talk about; she did not find the questions so relevant when filling out. The person with diabetes expressed afterward that “we covered other topics than usual which made it more personal–I think it was great” and was keen to keep using it. Another point the person with diabetes raised was that a question about medicine required for branch logic was irrelevant as the hospital should already have the information, which was true, and we began to prepopulate that item subsequently. This highlights the significance of iteratively testing every single item with users. The HCP evaluation confirmed that the person with diabetes was resourceful and knew what they wanted from the visit; the HCP also answered neutral (3) on whether the person with diabetes was better or worse prepared because of PRO.

#### Evaluation of the Acceptability of the PRO Evaluation Questionnaires

People with diabetes were able to fill out the evaluation questionnaires digitally without the need for support after each visit and expressed, in interviews, that the questions were relevant and easy to understand and fill out. We obtained complete evaluation data from both people with diabetes and HCP from all visits. This corroborated our initial finding from user-testing that they were suitable for use in routine care situations. As part of the formative design, the pilot evaluation questionnaires were revised based on our mixed-methods analysis and feedback from people with diabetes for use in a real-world study of how people with diabetes experience use of PRO [25]. Changes included alignment of items for people with diabetes and HCPs to evaluate interrater reliability, additional questions relating to perceived impacts of PRO on self-management versus HCP decision-making, role of the use of screens during the visit, emotional impact of PRO, and impacts of PRO on HCP’s work satisfaction and work stress. The final questionnaires were incorporated into a large-scale real-world study of the PRO diabetes tool in different health care settings [25].

## Discussion

### Principal Findings

The PRO tool helped improve the quality of the dialog and visit by facilitating the identification and prioritization of topics according to the needs of people with diabetes. In line with the extant PRO literature, the use of PRO helped introduce new talk topics, supported dialog about difficult psychosocial issues previously left unattended, and facilitated a more active role for people with diabetes [3,26,27]. People with diabetes felt better prepared for the visit, and in several cases, HCPs reported that people with diabetes appeared more ready to talk about difficult but clinically relevant issues, which has also been reported by other studies. The use of the PRO tool was feasible, acceptable, and facilitated a person-centered dialog in line with findings from other studies regarding the use of PRO to facilitate aspects of person-centered care [2,3,26-30].

### Meeting the Expectations of the People With Diabetes and Family Members

A common concern expressed by HCPs is that PRO questionnaires may raise unrealistic expectations for people with diabetes and that resources are not available to address topics raised by PRO [26,31,32], which could result in disappointment during the visit. All people with diabetes expressed that they received the care or support they needed from the visit; however, 1 person with diabetes expressed that the HCP did not sufficiently understand the person’s issues related to diabetes distress. All 12 people with diabetes in our study were positive about the PRO tool and the way the HCPs used it and did not report negative effects that they felt should prevent it from being a part of standard care. Although our combined analysis identified some issues identified by PRO that were not found relevant to act on, people with diabetes did not report any concerns related to lack of follow-up on PRO results or otherwise unfulfilled expectations.

Our finding that none of the people with diabetes expressed disappointment or unfulfilled expectations may be partly because the questionnaire had been specifically designed to only include items that were perceived as directly relevant for routine care by both people with diabetes and HCPs. Given the importance of expectation setting in the clinical use of PRO, we attempted to give instructions to people with diabetes about PRO carefully to avoid inadvertently raising unrealistic expectations.

### Fidelity and Use of Person-Centered Strategies

Our combined analysis indicated good fidelity related to the HCP’s use of the basic recommendations for person-centered use pertaining to the use of open questions to verify and clarify priorities of people with diabetes and ensure coverage of all key topics highlighted by them. The use of open questions and active listening was applied in all visits, and we found it to be an essential component that may be particularly important in the care of people with cognitive or language difficulties who may have difficulties completing the PRO questionnaire as intended. The PRO intervention did not include requirements for HCPs to use specific methods or tools for agenda-setting, structuring the visit, collaborative goal-setting, action-planning, and shared decision-making beyond the general recommendations for person-centered use of the tool. Therefore, this study provided a first opportunity for HCPs to try out the PRO tool in routine visits, identify key communication challenges, and begin to develop individual strategies on how to use the tool in an optimal way. The main challenges reported by the HCPs relate to how to structure the time and balance focus on clinical and PRO issues. An HCP expressed that it could be difficult to juggle tasks during the visit when people with diabetes had a high number of PRO topics, and an HCP found it difficult to review the many topics on the screen while maintaining a natural conversation. In contrast, and interestingly, the people with diabetes did not express concerns or problems related to the number of topics being identified and were overwhelmingly positive about the way the HCPs used the tool. We believe this may be partly because of the HCPs putting in an extra effort to ensure that people with diabetes had a good dialog experience despite the high number of flagged issues.

In a few cases, it was noted by HCPs that some flagged PRO topics, especially those related to symptoms, were not related to diabetes or were not relevant to act on in this visit; however, it was still necessary to review the topics as they were flagged on the PRO dashboard. The issue of not being able to act on especially generic PRO issues has also been reported by others [33]. Importantly, we found that people with diabetes appreciated the dialog on these topics, such as pain, sexual dysfunction, and sleep issues, as these issues were very distressing to them. Since the study, the PRO tool was further adjusted to include comorbidity information to facilitate the identification of symptoms unrelated to diabetes. Our results highlighted that the use of the PRO tool imposes significant demands on HCPs in relation to person-centered communication skills, self-efficacy for the use of PRO, and knowledge, as also highlighted by others [15,34]. Although the PRO tool was designed to be usable by diabetes HCPs with only minimal PRO-specific training, we believe that sparring with senior colleagues in developing individual person-centered strategies for the use of the PRO tool that suits each HCP’s style and clinical responsibilities will be important for optimal implementation and effectiveness. Clarifying each HCP team member’s role and responsibility in relation to the key PRO topics using frameworks such as 5A [35] may be important to support HCP adoption and self-efficacy for PRO use. PRO tools differ greatly in terms of how prescriptive the guidance is on how to apply PRO data for goal-setting and action-planning [30,36]. Further research is needed and underway to examine how different person-centered styles and communication strategies impact the use and impact of PRO [3,4,37,38].

### Benefits Related to Completion of the Questionnaire

People with diabetes expressed positive experiences related to the completion of the questionnaire. We found several factors that could potentially explain this. The completion of the questionnaire led to a reflective process that facilitated self- and disease-insight related to diabetes. During the design of the PRO tool, people with type 2 diabetes highlighted the potential for this questionnaire to help people with diabetes understand their situation and options for acting for their own health. On the basis of our results, this effect is likely important for a subgroup and dependent on diabetes duration, empowerment, and whether the person with diabetes is completing the PRO questionnaire for the first time. Filling out the questionnaire at home made people with diabetes feel confident that they would get to talk about their priority issues during the visit. This was very important as people with diabetes reported that they do not normally prepare for visits and often forget to ask about issues that are important to them. Furthermore, this appeared to have a potential impact on expectations and motivation related to participating in the upcoming visit.

### Benefits of a Broad Topic Coverage of the PRO Diabetes Questionnaire

The broad and balanced coverage of topics in the PRO diabetes questionnaire represents the result of an extensive iterative national multistakeholder participatory design process to achieve a core questionnaire acceptable and useful for both people with diabetes and HCPs in different care settings [3]. Our combined analysis found that a broad range of topics was relevant, usable, and perceived as beneficial either by the person with diabetes or the HCP. In addition to topics related to general life issues affecting diabetes, daily life with diabetes, self-management, and blood sugar regulation issues, people with diabetes expressed appreciation for the opportunity to speak about symptom distress related to pain and to address distress because of sexual dysfunction, which are topics not included in, for example, depression and diabetes stress–specific PRO tools [39,40]. Different PRO constructs have been shown to be suitable for different clinical purposes, such as dialog support, treatment decision support, screening, symptom monitoring, outcome evaluation, and collaborative care planning [12,27,41]. Some PRO constructs are mainly used for screening, such as hypoglycemia unawareness [42,43], cardiovascular symptoms [44], and depression [45], whereas items about, for example, medicine experience may be particularly useful for treatment monitoring and support [46,47]. Items about individual priorities and confidence in self-management can facilitate behavior change, collaborative care, and individualized self-management support [34,48,49].

Our study supports that it is feasible to use a PRO diabetes questionnaire with a very broad range of topics and that the questionnaire may generate both specific benefits related to each PRO construct as well as related to its use as an overview to facilitate a person-centered dialog [14]. We believe that the high acceptability and satisfaction with the tool by people with diabetes across diabetes type, age ranges, and different levels of disease progression may be partly because of the broad topic coverage, broad response options, and the use of branching logic. Although it was a focus area for the project if the questionnaire was burdensome, most people with diabetes appreciated the comprehensiveness of the questionnaire and expressed satisfaction with the experience of completing it as it *touched all the bases*. People with diabetes and HCPs did not report that any major topics were missing or that there were topics that were irrelevant. People with diabetes and HCPs confirmed that the scoring algorithms applied for the color-coding responses were suitable and fit for the purpose.

Further research is warranted to investigate the importance of a broad topic coverage to facilitate the potential therapeutic and empowering effect of self-completing the PRO. It is possible that PRO instruments that focus only on one or a few topics may not provide the same support for disease insight, care navigation, and active participation as instruments which provide a comprehensive 360°-review of diabetes issues

Another possible benefit related to the broad topic coverage, when compared with, for example, depression-focused diabetes screeners, is that people with diabetes who may be doing well emotionally are still given an opportunity to express other priorities and issues that affect them in relation to diabetes.

HCPs noted that an important benefit of the PRO tool was related to obtaining a structured overview of each person with diabetes’ overall situation, which was made possible by the comprehensive topic coverage. In a previous study, we found that there is a need to use a broad set of PRO outcome constructs to evaluate outcomes in diabetes as the needs of people with diabetes vary individually and change over time [16]. Recent standards for person-centered diabetes care also emphasize the significance of a comprehensive whole-person orientated evaluation of individual needs, preferences, and priorities [50].

Our PRO tool includes 5 items specifically related to depression and diabetes-related distress as it is important to legitimize and prompt dialog on these often insufficiently addressed issues [51,52]. Balancing items regarding these topics with items that reference daily life, self-management, symptoms, blood sugar regulation, medicine, and access to care may be important to set balanced expectations for visits. Although only an important minority of people with diabetes have mental health or emotional problems [53,54], it may be advantageous to invite people with diabetes to share their perspectives on all key aspects of care rather than only to express emotional symptoms.

### Initial Insights Regarding Evaluating the Public Health Impact Potential of the PRO Tool

This study provides initial insights on how to evaluate the potential public health impact of the PRO tool using the dimensions of RE-AIM (reach, efficacy, adoption, implementation, and maintenance) [9,55-57]. In terms of reach, all people with diabetes found the PRO tool usable and useful, which may reflect the fact that a large heterogeneous group of people with diabetes was involved in all stages of its development. In terms of efficacy, our study suggests that the tool has the potential to improve the experience of care and quality of diabetes visits in relation to person-centered principles. Future research is underway to examine the implications of the use of the tool for care quality, health, and cost outcomes. Regarding HCP adoption of the tool, HCPs were satisfied and interested in continuing the use of *DiaProfil*, and key barriers were found that were addressed by tool design and facilitation support. Further research is required to identify and address perceived barriers and facilitators to HCP adoption in a larger group of HCPs reflecting different disciplines and health care settings [14,25,58,59]. Implementation with adequate fidelity was established as possible by both physicians and nurses in our study. In terms of the PRO tool’s potential to become a permanent part of routine practice, that is, maintenance, we were encouraged by all people with diabetes expressing they felt that the PRO tool should be a part of standard of care without the need for further development.

### Overall Implications and a Conceptual Model

**Figure 3:**
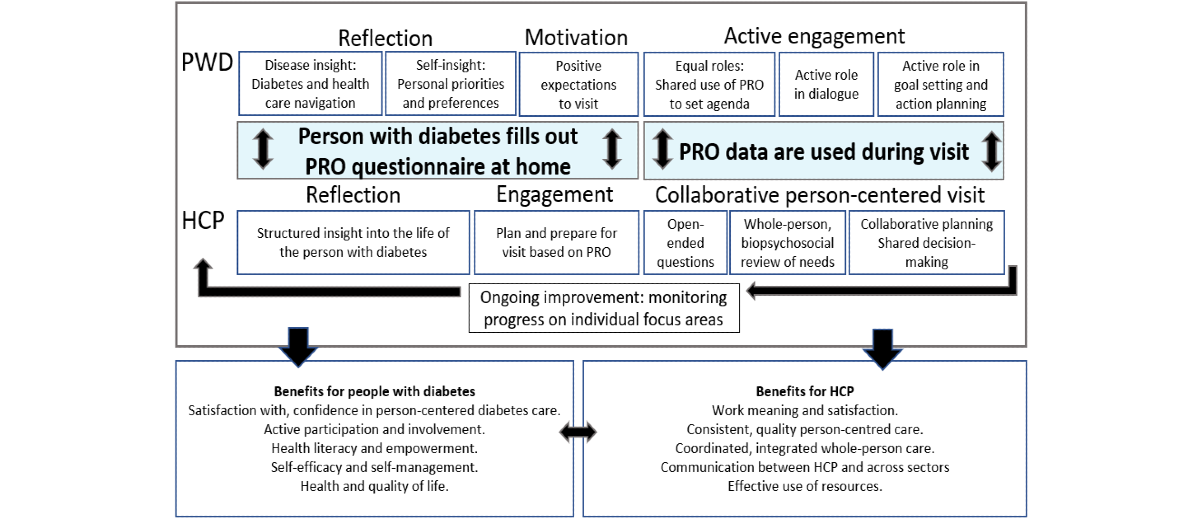
Conceptual working model for hypothesized processes and impacts for the use of the patient-reported outcome diabetes tool in a routine diabetes visit. HCP: health care professional; PRO: patient-reported outcome; PWD: people with diabetes.

Figure 3 shows an initial conceptual model to illustrate the hypothesized facilitators, barriers, and impacts related to the use of the PRO tool based on our combined data. This model will be expanded and improved as further and broader clinical and exploratory research is undertaken to incorporate a broader range of experiences, facilitators, barriers, and impacts.

The first stage of reflection and improved disease- and self-insight and motivation facilitated by the questionnaire completion by people with diabetes were found to be important to the study participants and suggest the potential for the intervention to facilitate diabetes-related empowerment [4,5,20].

Both people with diabetes and HCPs reported that people with diabetes were more actively engaged as a result of PRO. In this pilot study, HCPs used the PRO tool without detailed protocolized steps for agenda-setting, shared decision-making [60], goal-setting, or action-planning [61], so the visits involved a learning experience where they each identified relevant strategies to use.

The anticipated beneficial impact of the PRO tool for people with diabetes relates to confidence in diabetes management [62], self-efficacy [63] and empowerment [4,5,20,64], self-care behaviors [65], improvements to health and diabetes-related outcomes [16], and broader humanistic and societal outcomes [66] resulting from derived impacts.

HCPs appreciated gaining a structured insight into the lived experience of diabetes among people with diabetes, which facilitated reflection and allowed them to provide more personalized care. The PRO tool facilitated the introduction of daily life and psychosocial issues to achieve a comprehensive biopsychosocial review of the person with diabetes’ situation. As HCPs achieve self-efficacy for the use of PRO with their patients, there is a potential to experience improved work satisfaction and fulfillment. Given the utility of a wide range of topics, we believe the PRO tool has a particularly high potential for improving the individualization of treatments and use of a wider set of support resources in accordance with what can benefit individuals with diabetes the most, thereby potentially promoting better self-management, outcomes, and use of resources.

The *DiaProfil* tool was specifically designed to facilitate person-centered chronic illness care [67,68] and enable coordinated self-management and psychosocial support for people with diabetes across health settings. As part of the formative evaluation, we found that the PRO tool is helpful in raising awareness and use of a broader range of services such as diabetes education, social services, and other specialist care. Although the HCPs in this study used open-ended questions when using the PRO tool, this may not always be the case. Additional research is needed to evaluate how variations in the use of person-centered communication by HCP may impact the effectiveness of PRO and whether there may be differential impacts on vulnerable patient groups affected by factors such as low health literacy, low socioeconomic status, and social and psychological challenges [15]. Further research is specifically needed to examine the mechanisms for and potential benefits of using the PRO tool to strengthen navigation support for people with diabetes and coordination of care across sectors [69].

An important additional aim of the PRO diabetes tool is to improve care by monitoring outcomes that matter to people with diabetes for value-based care [16,25,70]. During the formative evaluation of the PRO tool, people with diabetes have been enthusiastic about the prospect of using the PRO tool evaluation to monitor progress and changes over time, and the functionality is built-in. Follow-up studies are underway to examine how and to what extent the use of the PRO data as outcomes can facilitate improvements in care.

### Limitations and Strengths

Our results should be examined while considering that this was a formative pilot study of the first viable version of the digital PRO tool, *DiaProfil,* before it was fully finalized for clinical use. This pilot study has important limitations: the study was not designed to provide empirical evidence for clinical effectiveness, and there was no attention control group, so it is not possible to rule out bias because of social desirability or effects related to study participation and added attention [71].

The small number of people with diabetes and HCPs limits the basis for generalization; even so, purposive sampling provided a good basis for examining experiences from a group of people with diabetes, which was diverse in terms of age, gender, type of diabetes, duration, treatment, and disease progression.

The 4 HCPs were previously involved in the design of the PRO tool, which limits the generalizability of their experiences. Further research is required to evaluate the adoption and implementation by a diverse group of PRO-naïve HCPs in different health care settings.

Despite these limitations, our use of purposive sampling, multi-informant, and mixed-methods data analysis provided an opportunity to show the robustness of our core findings. Further research is required and planned based on the detailed findings in the study to examine the impacts of, facilitators of, and barriers to effective and integrated standard use of our PRO diabetes tool on a larger scale in different health care settings [25]. Additional questions incorporated in our future study include questions relating to the perceived benefits of PRO among people with diabetes for self-management, care quality, and treatment outcomes, and HCPs’ confidence and skills in use of PRO in a person-centered manner and perceptions of impact of PRO on stress and work satisfaction.

### Conclusions

This is the first study to show the feasibility, acceptability, and perceived benefits of using the Danish PRO diabetes questionnaire and the *DiaProfil* tool in a routine diabetes care setting. We found that people with type 1 and type 2 diabetes in different age groups found the digital PRO diabetes questionnaire relevant, acceptable, easy to use, and as having good coverage of relevant topics. Both people with diabetes and HCPs found the digital PRO tool, *DiaProfil*, feasible, appropriate, and value-adding for use in routine diabetes care. The PRO tool helped improve the preparation and active engagement of people with diabetes and improved the quality of the dialog in line with the objectives of the PRO tool. Filling out the PRO questionnaire had a positive effect on a subgroup of people with diabetes by facilitating self-reflection and better visit preparation, which also contributed to readiness for talking about needed but difficult issues with the HCPs. HCPs were able to use the PRO tool in a person-centered manner and were satisfied with its functionality. Minor issues and challenges were identified, which were addressed as part of the participatory development process for the PRO tool related to some people with diabetes having a high number of PRO topics. Adjustments were made to the PRO tool to address these issues.

In conclusion, we found that our newly developed PRO diabetes tool, consisting of the national PRO diabetes questionnaire and the digital PRO tool *DiaProfil*, was acceptable to people with diabetes and HCPs, provided clinically useful dialog and decision support to HCPs, facilitated active participation of people with diabetes, and overall improved the perceived quality of diabetes care visits. Further large-scale, comparative, and controlled research is now warranted to examine its potential for large-scale implementation and positive public health impact.

## Abbreviations

HCP: health care professional
PRO: patient-reported outcome
RE-AIM: reach, efficacy, adoption, implementation, and maintenance
VBHC-PRO-DIA: Value-Based Health Care and Patient-Reported Outcome in Diabetes
